# Stable gene expression for normalisation and single-sample scoring

**DOI:** 10.1093/nar/gkaa802

**Published:** 2020-09-30

**Authors:** Dharmesh D Bhuva, Joseph Cursons, Melissa J Davis

**Affiliations:** Division of Bioinformatics, Walter and Eliza Hall Institute of Medical Research, Melbourne, VIC 3052, Australia; School of Mathematics and Statistics, Faculty of Science, University of Melbourne, Parkville, VIC 3010, Australia; Division of Bioinformatics, Walter and Eliza Hall Institute of Medical Research, Melbourne, VIC 3052, Australia; Department of Biochemistry and Molecular Biology, Biomedicine Discovery Institute, Monash University, Clayton, VIC, Australia; Department of Medical Biology, Faculty of Medicine, Dentistry and Health Sciences, University of Melbourne, Parkville, VIC 3010, Australia; Division of Bioinformatics, Walter and Eliza Hall Institute of Medical Research, Melbourne, VIC 3052, Australia; Department of Medical Biology, Faculty of Medicine, Dentistry and Health Sciences, University of Melbourne, Parkville, VIC 3010, Australia; Department of Clinical Pathology, Faculty of Medicine, Dentistry and Health Sciences, University of Melbourne, Parkville, VIC 3010, Australia

## Abstract

Gene expression signatures have been critical in defining the molecular phenotypes of cells, tissues, and patient samples. Their most notable and widespread clinical application is stratification of breast cancer patients into molecular (PAM50) subtypes. The cost and relatively large amounts of fresh starting material required for whole-transcriptome sequencing has limited clinical application of thousands of existing gene signatures captured in repositories such as the Molecular Signature Database. We identified genes with stable expression across a range of abundances, and with a preserved relative ordering across thousands of samples, allowing signature scoring and supporting general data normalisation for transcriptomic data. Our new method, *stingscore*, quantifies and summarises relative expression levels of signature genes from individual samples through the inclusion of these ‘stably-expressed genes’. We show that our list of stable genes has better stability across cancer and normal tissue data than previously proposed gene sets. Additionally, we show that signature scores computed from targeted transcript measurements using *stingscore* can predict docetaxel response in breast cancer patients. This new approach to gene expression signature analysis will facilitate the development of panel-type tests for gene expression signatures, thus supporting clinical translation of the powerful insights gained from cancer transcriptomic studies.

## INTRODUCTION

Measurements of transcript abundance are often used to infer the molecular phenotype of a biological system. Assays that capture such measurements vary in throughput, accuracy, and cost (1). Real time quantitative PCR (RT-qPCR) is a low throughput assay that requires smaller amounts of biological material, whereas whole transcriptome RNA-seq is a high throughput assay that requires a larger amount of RNA. In general, targeted RNA analyses tend to require smaller amounts of RNA are therefore applicable to samples where whole transcriptome analysis may not be feasible, for instance formalin fixed paraffin embedded (FFPE) samples (2). In a clinical setting, minimising the required amount of biological material is desirable as less needs to be acquired from a patient. Together with reduced costs, this makes RT-qPCR and other targeted sequencing technologies popular platforms for clinical RNA-based tests. The Oncotype Dx^®^ is a prognostic test that measures the abundance of 21 genes in breast cancer using RT-qPCR to compute scores that predict the risk of recurrence and guide chemotherapy (3).

NanoString's nCounter^®^ platform can provide moderate-throughput measurements of several hundred transcripts at a low cost, and this platform is used in the PAM50-based Prosigna^®^ breast cancer prognostic gene signature assay (4). This test first classifies samples into PAM50 subtypes and then uses these estimates to predict a risk or recurrence score. PAM50 subtypes are higher order molecular phenotypes of patient tumours that capture information on their molecular state. Lower order phenotypes that probe the microenvironment can be used to assess tumour infiltrating lymphocytes and potentially guide immunotherapies (5). Likewise, phenotypes that assess pathway activity may be used to predict drug sensitivity and consequently guide therapy (6).

In research, these phenotypes are routinely assessed using gene expression signatures such as those available in the molecular signature database (MSigDB) (7,8), and insights provided by them have been vital in developing our understanding of cancer. These signatures are often developed using perturbation experiments with an aim of probing transcriptomic data derived from systems where such perturbations are not possible (e.g. patient samples). Tens of thousands of gene signatures have been developed in the past however their widespread use in research and clinical contexts has been limited by the need for transcriptome-wide measurements which are expensive and unnecessary when only a small set of phenotypes need to be investigated. Additionally, these gene sets are not frequently used in samples that are formalin fixed and paraffin embedded (FFPE) as FFPE samples are not suitable for whole transcriptome RNA-seq. As a consequence, large and valuable collections of archival material cannot be used for RNA-seq based transcriptomics, but they may be amenable to alternative, low- to medium-throughput cost-effective methods like RT-qPCR or NanoString nCounter^®^. Supporting this approach, the testing of archival FFPE samples has shown targeted pathway methods to be the most reliable form of transcriptomic analysis for these samples (9). These use cases highlight the need for systematic methods for translating whole transcriptome derived gene expression signatures to a reduced measurement space that allow the potential exploration of archival samples and support the development of further targeted and cost-effective molecular phenotyping assays for use in research, pre-clinical and clinical settings.

A core requirement across all platforms is the need for control genes to normalise across sample measurements. Sample-to-sample variation can arise either form biological or technical sources. The effects of some technical variation can be minimised by normalising measurements against controls; this variation can arise from differences in total starting material, enzymatic efficiencies and in transcriptional activity between tissues or cells (10). More complex forms of variation such as batch effects can be corrected with sophisticated approaches that make use of control genes (11). Experimental spike-ins such as External RNA Controls Consortium (ERCC) spike-ins (12) can be used as control genes, however they do not experience the same sample preparation steps as endogenous RNA and therefore may not be the best representative reference (13–15). Historically, *ACTB* and *GAPDH* have been used as endogenous controls in RT-qPCR experiments, however, numerous studies have shown them to be differentially expressed across tissues (16–19). Alternatively, *housekeeping genes* that are assumed to be invariant across tissues due to their involvement in core cellular processes can be used as endogenous controls (20), however they have also been shown to vary across different tissues (10,16,21,22).

The availability of large transcriptomic datasets spanning numerous biological conditions has encouraged data-driven identification of reference genes. Vandesompele *et al.* (10) introduced the geNorm algorithm to select reference genes for microarray data by iterative elimination of the least stable gene. Key ideas they introduced were the use of multiple genes for normalisation, and evaluation of gene stability with respect to a putative set of stable genes. Reference genes for the NanoString nCounter® pan-cancer panels were selected by applying geNorm to the genotype-tissue expression dataset (GTEx). Krasnov *et al.* (21) used pan-cancer and normal RNA-seq data from the cancer genome atlas (TCGA) to prioritise reference genes for RT-qPCR experiments on tumour samples. Genes were prioritised such that they were differentially expressed between tumour and normal samples, had low variation as measured by the standard deviation, were associated with clinical parameters and had a high expression. Genes with many mutations, isoforms and pseudogenes were penalised. All information was weighted and collated using heuristic scoring functions. Lin *et al.* (22) have identified stable genes for normalisation single cell RNA sequencing (scRNA-seq) data. Using gamma-Gaussian mixture models for gene expression, they decomposed the lower expression spectrum of each gene into a gamma distribution. Next, they prioritised genes with a smaller gamma component, lower variation at the higher end of the expression spectrum, a smaller proportion of zero counts and lower differences between cell clusters. Their approach penalised genes with low expression across cell clusters.

Both data-driven approaches above defined stably expressed genes by computing multiple measures of stability and combining them into a single metric that can be used to prioritise stably expressed genes. A shared limitation of these gene sets was their derivation from a single dataset. Meta-analysis approaches generally produce robust results and have been widely used in differential expression analysis. As such, using multiple datasets to identify stable genes would produce a more robust prioritisation. Additionally, Krasnov *et al.* (21) proposed a cancer-specific set of stable genes but only used data from tumours to define their list. Most cancer-specific analyses are initially performed on cell lines and later translated to patients or patient derived xenografts. As such, genes used to calibrate these datasets need to be stable in both tumours and other experimental models.

In this study, we aim to address these limitations and improve the process of prioritising stable genes for use in the analysis of samples that may be derived from observational transcriptomic data (e.g. patient-derived samples and cell lines) where no specific perturbation has been applied. We compute a variety of stability measures across two diverse datasets and combine them using a meta-analysis method (23). A measure of outliers is included to ensure stability is maintained across as many samples as possible, and to allow usage of these genes in outlier-based analyses (24). The list of stably expressed genes we propose are comparable or better than other lists in terms of stability while possessing additional properties. Our list covers a wider range of expression values than previous studies and therefore may better capture variability towards the tails of the expression distribution. A novel property of rank preservation is observed whereby the relative ranks of stable genes are also preserved across samples. This additional information may provide better opportunities for some normalisation methods, and for rank-based analysis methods. Finally, we demonstrate how the list of stable genes along with information on their relative ranks may provide cost-effective opportunities to test for molecular signature enrichment, thus addressing one of the limitations of molecular phenotyping in the clinic. The lists we provide in this study may be used in a diverse set of applications, including, RT-qPCR normalisation, NanoString nCounter® normalisation, and RUV-based batch normalisation (11).

## MATERIALS AND METHODS

### Pre-processing datasets

Where count-level data were available, gene filtering was performed on log-transformed counts-per-million reads (logCPM) with subsequent trimmed mean of m-values (TMM) normalisation (25) and finally transformation to log-transformed reads per kilobase of transcript, per million reads (logRPKM). RNA-seq data from post-mortem samples were obtained through the Genotype-Tissue Expression consortium (GTEx). Some samples have undergone autolysis which results in poor RNA quality, therefore samples with autolysis scores >1 were excluded from the analysis. Pan-cancer RNA-seq data from The Cancer Genome Atlas (TCGA) were obtained as Subread-processed output from GEO (GSE62944). Samples with the phrase ‘carcinoma’ in their annotation were considered carcinomas and were used to derive our stable gene list. TCGA breast cancer data were downloaded and processed using an alternate pipeline described in a R/Bioconductor-based workflow (26). This data was used to assess the impact of processing pipelines on putative stable genes. Cancer cell line encyclopedia (CCLE) samples were classified as carcinomas similar to TCGA samples. CCLE data downloaded using the PharmacoGx R package (27) were used in this analysis. Finally, genes with a logRPKM, log-transformed transcripts per million (logTPM), logCPM, or log-transformed fragments per kilobase of transcript, per million reads (logFPKM) of less than 1 across more than half of the samples were filtered out from each dataset due to low abundance. Filtering to remove genes with low abundance was performed independently for carcinoma and non-carcinoma samples.

Processed level 3 data from the Connectivity Map (CMap) project (28), representing a large collection of gene and compound perturbations applied to 76 cell lines was downloaded from the Gene Expression Omnibus (GEO) from the accession GSE92742. A compendium of compendium of 10 datasets covering nine cell lines combined and corrected for batch effects by Foroutan *et al.* (29) was downloaded from the University of Melbourne's figshare (DOI: 10.4225/49/5a2a11fa43fe3). Gene identifiers from both datasets were converted from Entrez IDs to gene symbols using annotations from the org.Hs.eg.db R package (v3.11.4).

Sequencing quality control consortium (SEQC) RNA-seq data were obtained from the seqc R/Bioconductor package. RNA-seq measurements from Illumina instruments at the Australian Genome Research (AGR) centre processed using the RefSeq annotation were used in this study. RT-qPCR measurements from the PRIME-qPCR protocol were obtained from GEO (GSE56457).

Transcriptomic measurements from primary biopsies of 24 breast cancer patients treated with docetaxel were downloaded from the GEO from the accession GSE6434. Processed microarray data were downloaded, and log transformed. Probes representing the same gene symbol were combined using the average.

### Computing metrics of variability

Four metrics of variability were computed for each gene within each dataset:

- The median absolute deviation (MAD): a rank-based measure of variation. For gene expression measurements ${X_1},{X_2}, \ldots ,{X_n}$, where ${X_i}$ is the expression of gene $i$ across $m$ samples, the MAD is calculated as ${\rm{MAD\ }} = {\rm{\ median}}( {| {{X_i} - \bar{X}} |} )$ where $\bar{X}{\rm{ = \ median}}( {{X_i}} )$.
- Shannon's entropy: a measure of information content for a variable. The Shannon entropy is computed using the distribution of the data. We estimate the distribution by discretising gene expression measurements into bins of equal width using the entropy R package. The number of bins was computed as the square root of the number of samples and bins were defined using the expression distribution of the entire dataset. Shannon's entropy for gene *i* is then computed as ${\rm{I\ }} = \ - \mathop \sum \limits_{i\ = \ 1}^k {\rm{P}}( {{x_i}} )\log {\rm{P}}( {{x_i}} )$ where $P( X )$ is the probability mass function and *k* is the number of bins.
- The outlier sum statistic: a metric quantifying the presence of outliers. Outliers are defined as observations where transcript abundance is either greater than the sum of the third quartile and the interquartile range (IQR), or less than the difference of the first quartile and the interquartile range ($x < {q_{0.25}} - {\rm{IQR}}$ or $x >{q_{0.75}} + {\rm{IQR}}$). The outlier sum statistic is then the sum of absolute value from median-centred outliers.
- *F*-statistic from a one-way ANOVA test on source tissue to assess group-wise differences.

R code used to compute these metrics and combine the results is available in additional file 3.

### Stability scores for gene sets

We repurposed singscore to compute gene set stability scores instead of enrichment scores. Genes were ranked based on their stability across all TCGA carcinoma samples and CCLE carcinoma-derived cell lines. We then created a single pseudo-sample with genes ranked on stability and computed uncentred scores for this pseudo-sample against gene sets using the R/Bioconductor package singscore. The resulting scores are each in the range of [0,1] with 1 indicating perfect gene set stability relative to all assessed genes. Gene sets were downloaded from MSigDB v5.2 (8). We discarded gene sets where fewer than 10 member genes had been assessed for stability in our study. The remaining gene sets were scored for stability using the approach described.

### Computing gene set scores using stable genes

The *stingscore* approach to scoring gene sets using stable genes is implemented in the R/Bioconductor package singscore (v1.8.0). Expression data can be ranked against stable genes by passing a set of stable genes to the rankGenes() function using the stableGenes argument. Passing the rank matrix to the simpleScore() function automatically invokes the *stingscore* implementation.

### Deriving a docetaxel gene signature using CMap

Two level 4 processed *z*-score profiles representing docetaxel treatment of MCF7 cell lines were downloaded from https://clue.io/ (command: /sig ‘docetaxel’) and processed in R using the R/Bioconductor package cmapR (v1.0.0). The two profiles were combined using the moderated *z*-score approach (28). Genes with a z-score greater than 1.2816 (90th percentile of standard normal distribution) were considered as up-regulated and formed the docetaxel gene signature. Only up-regulated genes were selected to form the signature as from past experiences, they tend to carry a stronger signal (26).

## RESULTS

### Selecting stably expressed genes

In our study we have explored expression stability of genes relative to other genes. As such, variation of a gene across samples could be biologically significant, but it would be considered stable if it is less variable than other genes. In effect, like previous studies, we identified genes with a smaller dynamic range relative to other genes. Two cancer datasets representing different cancer models were used to identify stable genes: The Cancer Genome Atlas (TCGA) pan-cancer tumour data and Cancer Cell Line Encyclopedia (CCLE) cell line data (27,30). As the transcriptome of solid tumours are clearly distinct from those of liquid (haematological and lymphoid) malignancies, we have focused on identifying stable genes in solid tumours for this study. Thus, only carcinomas from TCGA and carcinoma-derived cell lines from the CCLE dataset were used to identify stable genes. Using multiple variability measurements across diverse datasets ensured a robust selection of stable genes.

**Figure 1.**
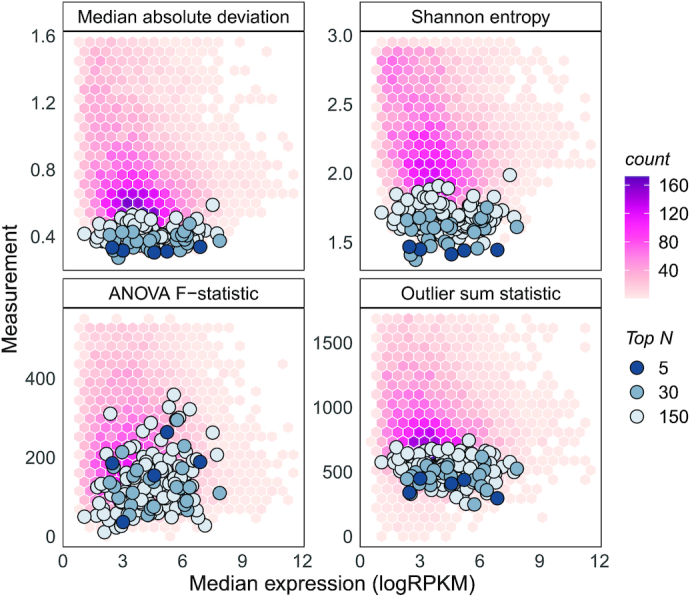
Stability metrics for stable genes are minimised on TCGA PanCancer carcinomas. The background distribution represents metrics for all genes. Top 5, 30 and 150 stable genes are overlayed on each plot. These genes have lower median absolute deviations, Shannon's entropy and outlier sum statistics. They have relatively lower between-group differences as signified by the F-statistic from the one-way ANOVA test on tissues. Stable genes tend to cover a wide range of the expression spectrum (widely distributed median logRPKMs).

For each gene, we calculated the median absolute deviation (MAD), Shannon's entropy, the outlier sum statistic, and the *F*-statistic from a one-way ANOVA analysis - on either the tissue of the primary tumours or the tissue of origin for cell lines. The first three metrics quantify variability of gene expression while the last quantifies between group differences in abundance. Ideal stable genes would be invariant to tissue types. Hexbin plots in the background of Figure 1 show distributions of these measurements for TCGA carcinomas. These four quantities were measured for all genes across both datasets, resulting in eight measurements of variation.

Considering each of the eight variation metrics as separate stability analyses, we can use meta-analysis approaches to prioritise stable genes. We used the *product of ranks* rank aggregation method to combine stability information gained from the measurements (23). Briefly, genes were ranked in ascending order of each variability measurement independently resulting in eight *rankers*. These rankers were then combined using the product function to produce a product of ranks statistic for each gene. Genes missing in either dataset were discarded as their product of ranks could not be computed. A schematic of this approach is presented in the supplementary material (Additional file 1: Supplementary Figure S1). The proposed list of stable genes can be accessed using the getStableGenes() function in the R/Bioconductor package singscore (v1.8.0). The top 5, 30 and 150 stable genes prioritised using this approach are overlayed on TCGA metric distributions in Figure 1. It is evident that the product of ranks approach prioritises genes that minimise most measures of variation. The *F*-statistic from the ANOVA analysis is higher for proposed stable genes. Variability measurements were plotted against the median expression value of each gene in Figure 1, demonstrating that our set of top stable genes covers a wide range of expression values.

### Comparison against other lists of stable genes

We used 14 independent datasets to assess the validity of our prioritisation of stable genes. These data are listed in Table 1 with details on the number of samples, groups and measurements, and the type of data or measurement. They are derived from tumour samples, cancer cell lines, normal tissue, and primary cell lines; with RNA-seq, proteomic and CAGE-seq measurements. Pre-processing was different for some of the data with RNA-seq and CAGE-seq measurements summarised as either TPM, CPM or FPKM/RPKM.

**Table 1.** Datasets used to identify stably expressed genes and assess their stability. Datasets are grouped based on the projects they were sourced from and are annotated for the type of dataset, number of samples, number of biological groups and the measurement used (TPM – transcripts per million, RPKM/FPKM – reads/fragments per kilobase per million or CPM – counts per million). Original studies that produced the dataset are cited along with the study where the processed version as downloaded. Processed versions of datasets marked by asterisk were downloaded from the human protein atlas www.proteinatlas.org

Project	Dataset	Type	Measurement	Number of samples	Number of groups	Citations
**TCGA**	TCGA carcinomas	Pan-cancer tissue	FPKM	7310	13	(26,44)
	TCGA other	Pan-cancer tissue	FPKM	1942	10	(26,44)
	TCGA BRCA CPM	Breast cancer tissue	CPM	1077	6	(26,44)
	TCGA normal	Normal tissue	FPKM	718	20	(26,44)
**CCLE**	CCLE carcinomas	Pan-cancer cell line	RPKM	581	19	(27,30)
	CCLE other	Pan-cancer cell line	RPKM	348	15	(27,30)
**HPA**	HPA tissue	Normal tissue	TPM	43	43	(45)
	HPA cell line	Normal cell line	TPM	64	64	(45)
	HPA blood sample	Blood	TPM	109	19	(45)
**CPTAC**	CPTAC TCGA colon	Colon cancer tissue	MS/MS intensity	95	-	(46)
**FANTOM**	FANTOM CAGE tissue*	Normal tissue	TPM	45	45	(47-49)
**GTEx**	GTEx (v7)	Normal tissue (post-mortem)	TPM	8462	29	-
**Other**	Daeman *et al.* breast cell lines	Breast cancer cell line	RPKM	64	4	(31,35)
	GSE60424 sorted blood	Blood	RPKM	28	7	(38,50)
	Monaco *et al.* blood*	Blood	TPM	30	30	(45,51)
	Schmiedel *et al.* blood*	Blood	TPM	15	15	(32,45)

**Figure 2.**
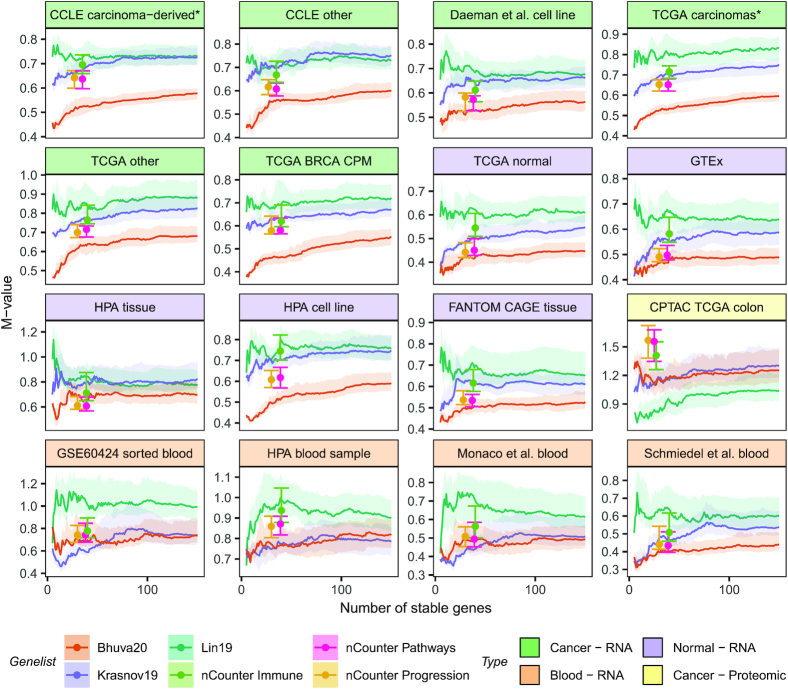
The proposed list is more stable than other lists across most of the 14 datasets used to assess stability. Our prioritisation of stable genes is compared against those from Krasnov *et al.* (2019) and Lin *et al.* Stability is compared against fixed lists used in the NanoString nCounter^®^ PanCancer gene panels. Our list results in a better prioritisation of stable genes in cancer patient and cell line datasets. Additionally, our list is more stable in normal samples. Different summarisations of transcriptomic measurements (RPKM, TPM and CPM) do not affect stability. Stability of genes proposed in all lists is not preserved in blood, likely due to the distinct biology of blood. Additionally, our list like most others was identified on solid tissue data, therefore, did not capture stability in blood. Genes proposed by Lin *et al.* tend to be more stable in proteomic data. This list was proposed for scRNA-seq data which suffers from the same problem of missing values as proteomic data therefore would work well with proteomic measurements.

We evaluated our prioritisation of stable genes on these datasets and simultaneously compared it against other stable gene lists or prioritisations. Specifically, we compared our rankings against those from the ordered lists of Lin *et al.* (22) and Krasnov *et al.* (21). We also compared stability against the set of stable genes used for normalisation in the NanoString nCounter^®^ PanCancer pathways, PanCancer immune profiling and PanCancer progression gene expression panels. These panels have 40, 40 and 30 stable genes, respectively. To enable comparison of both discrete and prioritised lists of stable genes, we computed *M*-values proposed in the geNorm method (10). *M*-values are computed to capture the variability of each gene relative to every other gene in a putative set of stable genes. Thus, adding a gene to the set of stable genes will alter the *M*-value for all other genes in the set. We computed the median *M*-value and the interquartile range of *M*-values for a given set of stable genes. These values were computed for sets of size 5–150 for prioritisation lists such as ours. These quantities are shown in Figure 2 where the median *M*-value is represented as either a point or a line, and the interquartile range as error bars/bands. Lower *M*-values indicate better stability.

As shown, our list outperforms others in stability measures across the datasets used for their derivation. Additionally, our list outperforms other lists in all TCGA datasets, including non-carcinomas, normal tissue samples and the breast cancer cohort with gene expression measured in CPM. Interestingly, all lists of stable genes tend to be more stable in normal TCGA samples than in cancer samples as evident by the relatively lower *M*-values. We note that magnitudes of M-values should not be compared between datasets if data are drawn from different underlying distributions. It is a valid comparison for RPKM-level data from TCGA as they were prepared and processed similarly. Our genes are more stable than other lists across cancer datasets, including CCLE non-carcinoma cell lines and Daemen *et al.* (31) breast cell lines. Additionally, our genes are relatively more stable across normal tissue and primary cell lines except for the human protein atlas tissue data where the NanoString nCounter® PanCancer Pathways and PanCancer Progression panel genes are more stable compared to our lists of equivalent size. Control genes for the NanoString nCounter® panels were selected by optimising M-values in the GTEx data, yet our lists of equivalent size are more stable in those data. We also showed stability of our list on data generated using a different protocol for measuring transcript expression, CAGE-seq.

The most distinct datasets were the blood RNA-seq and cancer proteomic datasets. None of the lists of stable genes are clearly more stable than the others in blood RNA-seq data. The top 50 stable genes from Lin *et al.* (22) tend to be more stable in the GSE60424 sorted blood data, but the trend does not hold across other blood datasets. Our list outperforms other lists in the blood data generated by Schmiedel *et al.* (32). Interestingly, control genes used in the NanoString nCounter® PanCancer Immune panel tend to be less stable in blood data than our stable genes and those proposed by Krasnov *et al.* (21). Finally, the list by Lin *et al.* (22) is the most stable in the label-free quantification proteomic dataset from the CPTAC project. Overlap analysis between the different lists showed that there was little overlap between our list of stable genes and other lists (Additional file 1: Supplementary Figure S2). As such, the list of stable genes we propose is relatively novel. Additionally, the set of reference genes commonly used across many gene expression panels (gathered by Krasnov *et al.* (21)) have the little overlap with genes identified from data-centric approaches.

Though our list of stable genes was identified using observational samples such as patient samples and unperturbed cell lines, we investigated their stability in perturbation experiments which are often performed on experimental models such as cell lines. We assessed stability using data from two perturbation experiments: The Connectivity Map project (CMap) where 76 cell lines were perturbed using thousands of chemical and genetic perturbagens, and a compendium of datasets where a large phenotypic shift was induced by stimulating cell lines with TGFβ. Stability analysis using *M*-values reveals that our list of stable genes outperforms other prioritisation-based lists, however, it is less stable than the set of control genes used in NanoString nCounter^®^ PanCancer pathways and progression panels (Additional file 1: Supplementary Figure S3). Expression measurements from the CMap project are measured using the L1000 assay which directly measures the expression of only 978 genes and infer the expression of more than 10000 other genes therefore we also investigated the accuracy with which genes within each list were measured. This analysis shows that control genes in the NanoString nCounter® panel are measured with slightly greater accuracy than other lists of similar sizes (Additional file 1: Supplementary Figure S3). Since the data from Foroutan *et al.* (29) only contained two groups (TGFβ-stimulated and controls), we performed t-tests between these groups and used the resulting t-statistic as a measure of variability. Our analysis shows that top-ranking stable genes in our list are more stable than other genes, however, stability between all lists is comparable when larger gene sets are assessed (Additional file 1: Supplementary Figure S4).

### Functional composition of stable genes

Next, we investigated the functional role of genes with stable expression. Since these genes were expressed at stable levels across a wide range of tissues, we suspected they may be involved in essential processes. To test this hypothesis, we used the list of essential genes identified in the DepMap project (33,34) using CRISPR knock-out screens across a variety of cell lines. The DepMap project identified 2164 genes that were essential for survival. We evaluated their occurrence in our list of stable genes and noticed that for lists of any size, approximately half the stable genes were essential for survival. Similarly, ∼65% of the stable genes proposed by Lin *et al.* (22), ∼35% of the genes proposed by Krasnov *et al.* (21) and ∼40% of the genes in the NanoString nCounter^®^ panel were essential for survival (see Additional file 1: Supplementary Figure S5). This analysis suggested that genes essential for survival tend to be stably expressed and the set of essential genes may be better a biologically motivated set of stable genes than the set of housekeeping genes.

Since stable genes were a mix of essential and non-essential genes, we further characterised our list using gene ontology (GO) enrichment analysis. Our aim was to evaluate enrichment accounting for the prioritisation of stability in our list. We adapted singscore (35) to achieve this and computed stability scores for gene sets derived from gene ontologies and KEGG pathways. Gene sets associated with RNA processing (GO:0006396, GO:0008380), the spliceosome complex (GO:0005681), mRNA metabolic process (GO:0016071) and RNA binding (GO:0003723) were some of the gene sets enriched with stable genes (see Additional file 1: Supplementary Figure S6) and had varying proportions of essential genes (50–75%, see Additional file 2). Similar analysis of KEGG pathways as gene sets revealed the spliceosome pathway (hsa03040) to be the most stable. The epithelial mesenchymal transition gene set from the hallmarks set of MSigDB was the most variable (least stable). Stability scores of all MSigDB gene sets can be further explored using the interactive plot available in Additional File 2.

### Relative ranks of stable genes are preserved across samples

The perfect stable gene would be completely invariant under all conditions. Given two such genes expressed at different abundances, we would observe that across all samples, the gene with higher expression would always be ranked higher than the other gene. This relationship would slowly dissolve as these genes become more variant or the difference between their average expression reduces. As such, given a set of stable genes, their ranks based on gene expression would be consistent so long as the genes were stable. We identified this effect for the highest and lowest expressed genes in our top 5 stable genes, *RBM45* and *HNRNPK* respectively. *RBM45* expression was lower than *HNRNPK* expression for all samples across all 15 RNA-seq datasets. This strong rank preservation is in part attributed to the large difference in expression between the two genes.

**Figure 3.**
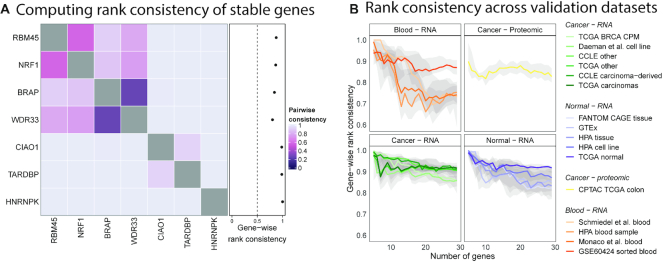
Expression ranks of stable genes are preserved relative to each other. (**A**) Pairwise rank consistency measured as the proportion of samples where the expected ranks learned from the training datasets matches the observed rank. The gene-wise rank consistency is then the average of a gene's pairwise consistencies. The gene-wise consistency of HNRNPK is 1 indicating that its rank relative to the six other stable genes being considered is as expected (higher than the other genes in all samples). (**B**) Median gene-wise rank consistencies plot for stable genes from lists of sizes 5–30. Bands represent the inter-quartile range. Rank consistency is low in blood datasets due to reduced stability.

Expression-based ranks for genes were computed using the product of ranks meta-analysis approach. We ranked stable genes according to their median expression in the datasets used to identify them (TCGA carcinomas and CCLE carcinoma-derived cell lines). Using the rank of median expression in each dataset as an individual *ranker*, we computed the product of ranks to determine the expression-based rank of each gene. As such, information on the expected ranks based on abundance is added to any discretisation for our list of stable genes. Next, we evaluated rank preservation for stable gene sets of sizes 5–30 across all datasets. For each pair of genes within a set of stable genes, we first computed the pairwise rank consistency as the proportion of samples where the order of gene expression matched the expected order. Then, for each gene, we defined the gene-wise rank consistency as the average of its pairwise consistency with all other genes in the set. Like the *M*-value, the gene-wise rank consistency of a gene is defined relative to other genes in the gene set. Figure 3A shows the pairwise rank consistency measurements for the top 7 stable genes along with their gene-wise rank consistencies in TCGA carcinomas.

We computed gene-wise rank consistencies of genes in stable gene sets of varying sizes across the 14 validation datasets. Figure 3B shows the median of computed gene-wise rank consistencies along with the interquartile range for each set of stable genes. As expected, ranks are preserved in datasets used to derive stable genes and their expected order. Ranks are preserved strongly in cancer datasets and in normal tissue/cell line datasets with a slightly higher preservation in the former. Rank consistency within blood datasets is generally lower compared to all other datasets. Larger sets of stable genes tend to be strongly preserved in TCGA normal samples. Rank preservation is observed in the CPTAC colon proteomic dataset, though not as strongly as with transcriptomic dataset.

### Computing gene expression signature scores using a reduced number of measurements

We previously developed a method, singscore, to score individual samples against gene set signatures using transcriptomic data and showed that these scores can assist in assessing the molecular phenotype of tissues and cell lines (35). Though the method has been applied in diverse scenarios in an exploratory context (26,35–38), the potential for clinical translation is limited by a requirement for transcriptome-wide measurements. Singscore ranked genes based on their transcript abundance, computed the mean of expected up-regulated genes in the case of an up-regulated gene signature, and normalised the mean to produce a signature score. Higher scores indicated concordance with the gene signature. Transcriptome-wide measurements were only required to rank genes in the signature, thereby providing context on how highly/lowly expressed genes in the signature were relative to all other genes.

**Figure 4.**
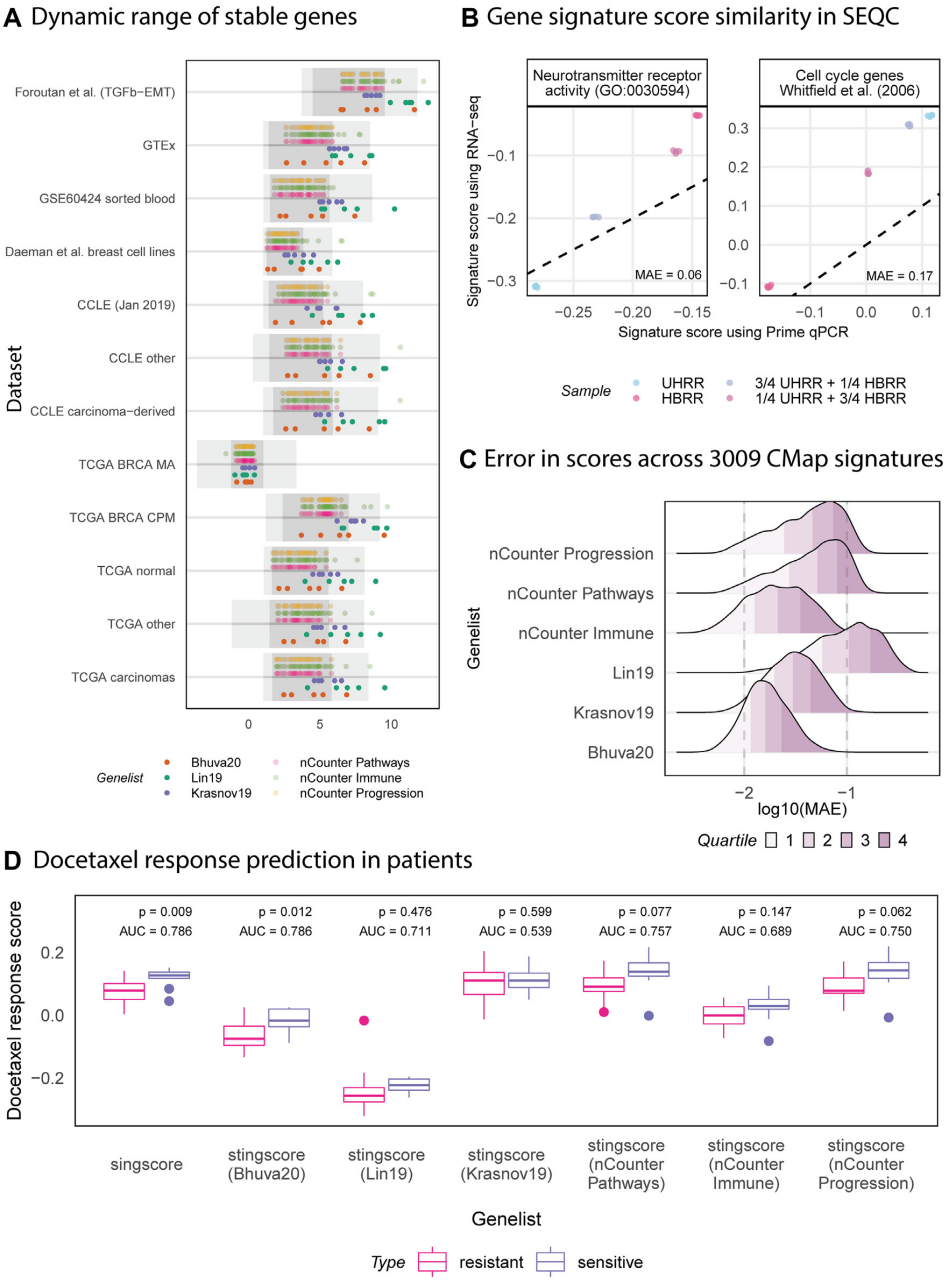
Scores computed from transcriptome-wide RNA-seq measurements are comparable to scores computed from a small panel of genes and our set of stable genes. (**A**) Median expression of the top 5 stable genes from each of the prioritisation-based lists are plot against the interquartile range (dark grey) and 1%-99% range (light grey) within each of the training and validation datasets. Our list tends to have a wider dynamic range than other lists across most datasets and our genes are well spaced across this range, therefore, they are more suitable for scoring using stingscore. (**B**) The brain RNA sample (HBRR) scores highest for the brain-specific signature (neurotransmitter receptor activity) with scores reducing as the proportion of brain RNA reduces. The inverse is observed with a cell cycle gene signature which should be more active in cancers. Scores computed using singscore are comparable to those computed using stingscore and our top 5 stable genes. (**C**) Difference between scores computed with singscore and stingscore using different sets of stable genes measured as the mean absolute error (MAE). Quartiles of MAE are coloured. Top 5 stable genes are selected for prioritisation-based lists (Lin *et al.* (22) and Krasnov *et al.* (21)). Scores are computed for 3009 gene signatures across a total of 75012 expression measurements from the connectivity map project (CMap). (**D**) Scores computed with singscore and stingscore using different sets of stable genes are used to discriminate docetaxel sensitive (*n* = 10) patients from resistant (*n* = 14). Patients are scored using a docetaxel signature derived from CMap (52 up-regulated genes). Area under the receiver operating characteristic curve (AUC) are computed along with *P*-values from a *t*-test. Singscore and stingscore with our set of stable genes provide the best separation of samples.

The rank preservation property of stably expressed genes can be used to calibrate measurements within a sample, thus providing an appropriate context to evaluate the relative expression levels of all other genes. Stable genes allow relative rank estimation of genes without the need for transcriptome-wide measurements. The relative transcriptome-wide rank of any gene can be interpolated given a set of stably expressed genes that are equally spaced on the expression spectrum and that span the entire range of expression values. Figure 4A shows that our set of stable genes cover a wide range of the gene expression spectrum and are approximately evenly distributed, therefore, may be used to approximate the ranks of other genes without the need for transcriptome-wide measurements. We approximated the unit normalised ranks of genes using a simple approach (see illustration in Additional file 1: Supplementary Figure S7). For any given set of stable genes, the rank of a gene was approximated as the number of stable genes with expression values lower than its expression. Unit normalisation was performed by dividing this number by one plus the total number of stable genes in the stable gene set. The signature score of a signature consisting of up-regulated genes was simply the average of their unit normalised ranks. Further normalisation would not be required since ranks were already normalised. A similar procedure would be applied for signatures consisting of down-regulated genes; the only difference being the inversion of ranks (1 – unit normalised rank). Scores using both up- and down-regulated genes were centred around 0 by subtracting the median score (0.5) from them, thus ensuring score were in the range [–0.5, 0.5]. Additionally, signatures composed of both up- and down-regulated genes with unknown direction can be scored using an approach similar to singscore (35).

We compared scores computed using the original singscore with those computed using our new method, *stingscore* (stable singscore), that only requires measurements of genes in the transcriptomic signature and a few stable genes. Samples from the SEQC/MAQCIII project were used to compare scores computed using the different approaches on different measurement platforms (39). The SEQC/MAQCIII project had measurements for four samples: universal human reference RNA sample (UHRR), the human brain reference RNA (HBRR), a mixture containing ¼ UHRR and ¾ HBRR, and a mixture containing ¾ UHRR and ¼ HBRR. HBRR was extracted from multiple brain regions of multiple patients and UHRR was extracted from different tumour tissues of different patients. Measurements of all samples were taken using RNA-seq and RT-qPCR. We scored all samples against a brain-specific neurotransmitter receptor activity gene signature (GO: 0030594) and a cancer-associated cell cycle signature (40). Scores were computed using two approaches: using singscore with transcriptome-wide RNA-seq measurements and using *stingscore* with RT-qPCR measurements of genes in the signatures (67 and 44 genes for the brain-specific and cancer-associated signatures respectively) and the top five stable genes identified in our analysis. Since RT-qPCR measures cycle threshold (C_t_) values, we ranked genes using 1/C_t_. By sampling from the RT-qPCR measurements, we replicate a clinical setting where signatures are evaluated on samples. Scores computed from transcriptome-wide RNA-seq measurements are highly correlated with those from RT-qPCR measurements of signature genes and stable genes (Figure 4B). Despite the high correlation between scores computed using the different approaches, there is a noticeable yet variable offset in scores. Relevant biology is recapitulated by scores computed using *stingscore*, with HBRR scoring the highest for the brain specific gene signature followed by samples with decreasing amounts of HBRR and finally UHRR having the lowest score. The inverse is noticed with a cell cycle signature which we would expect to be more active in cancers than normal tissue.

Analysis of the SEQC data demonstrated the ability of stingscore to reproduce scores using a targeted transcriptomic panel measured using assays such as RT-qPCR. We then wished to demonstrate reproducibility of scores across a wider set of conditions and datasets. Since matched RNA-seq and RT-qPCR data with the stable genes of interest are rarely available, we used a down sampling approach to simulate targeted transcript measurements from whole-transcriptome data where only genes in a given signature along with a set of stable genes would be used to score samples. Gene signatures from the CMap project were used to demonstrate score comparability across a variety of conditions. Of the hundreds of thousands of signatures generated in phase I of the CMap project, only those signatures that were derived from more than 10 experiments and had matching transcriptomic measurements were used. This resulted in 3009 signatures, each with more than 10 transcriptomic experiments across a variety of cell lines (total of 75 012 experiments). For each signature, we then scored the samples used to generate the signature against the signature itself using both singscore and stingscore. Since samples were scored against the signature they represent, they should be associated with the relevant biology and therefore are true positives. The difference in scores across samples were summarised using the mean absolute error (MAE) for each signature. Scores were computed using all lists of stable genes, with the top 5 stable genes used from the prioritisation-based lists. Figure 4C shows the distribution of errors, along with the quantiles from scores computed using the different sets of stable genes. It is evident that our list of stable genes produces smaller errors in scores computed with stingscore against those computed using singscore with transcriptome-wide measurements. Control genes in the nCounter^®^ PanCancer Immune panel tend to have smaller errors compared to the other nCounter^®^ panel control genes. It is difficult to perform further comparisons between nCounter^®^ control genes and prioritisation-based genes due to size differences in these lists.

Finally, we performed a very specific and clinically relevant evaluation of stingscores using the different sets of stable genes. Using transcriptomic measurements from core biopsies of 24 breast cancer patients (GSE6434) (41), we attempted to predict response to neoadjuvant docetaxel treatment using gene signatures derived from the CMap project corresponding to docetaxel treatment of MCF7 cell line. Response for these patients was known with 10 responding to, and 14 resisting taxane treatment. Patients were scored against the docetaxel signature using both singscore and stingscore with the top 5 genes from each of the prioritisation-based gene sets, and with all genes from the nCounter® PanCancer panels. The *t*-test was performed on scores from each method to determine the ability of scores to discriminate resistant and sensitive patients. Figure 4d shows the distribution of scores computed for resistant and sensitive patients along with *P*-values from a *t*-test. Area under the receiver operating characteristic curve (AUC) was also computed to determine classification performance. The results show that singscore provides the best separation of patients based on response, however this requires transcriptomic data, and it is closely followed by stingscore combined with our set of stable genes. The list from Lin *et al.* (22) also performs moderately well, whereas stingscore combined with the list from Krasnov *et al.* (21) provides almost no distinction between the two groups. Scores computed using the nCounter® PanCancer pathways and progression panels discriminate samples based on response however their performance is lower than when using our top 5 stable genes.

## DISCUSSION

Genes with stable expression have frequently been used for data normalisation including correction of batch effects (10,11). In this study, we derive a new set of stable genes for application in cancer transcriptomic analyses and demonstrate their additional use for targeted molecular phenotyping beyond data normalisation. Targeted molecular phenotyping is particularly useful in the analysis of archival tissue where rich clinical information is present but the preservation techniques limit extraction of high-quality RNA and consequently RNA sequencing. These samples are suitable for other smaller scale measurement platforms such as NanoString® and RT-qPCR. Thus, exploration of strategies such as *stingscore* that move from reliance on a whole transcriptome of measurements to the measurement of tens or hundreds of transcripts will support the analysis of these valuable and vast historical collections of tissues.

We formulated the identification of stable genes as a meta-analysis problem and used the product of ranks approach to combine multiple stability metrics across two datasets. Our approach provides flexibility in adding/removing variability metrics and datasets. Since the product of ranks requires all rankers (the test metrics) to provide a complete ranking, we had to discard genes that were not measured and consequently not ranked in either dataset. While this loss was relatively small for integration of two datasets, it would be more pronounced if multiple datasets were used. Other methods that allow rankers to provide partial rankings might be used (42), but in the specific application of identifying a general set of stably expressed genes, it makes sense to limit our analysis to genes that can be measured reliably.

Our set of stable genes covered a wide range of expression values (see Figure 1) even though this property was not selected for explicitly. This is likely a result of using multiple measures of variation which reduced biases resulting from the mean-variance relationship often observed in RNA-seq data (43). A wide range of expression values is desirable for normalisation, as such genes capture variation at the tails of the gene expression spectrum. A wide range is also important for the evaluation of gene expression signatures that capture down regulated genes, where we expect low abundance. Our selection of stable genes also minimises between-group differences by minimising the *F*-statistic from a one-way ANOVA test on groups. Stable genes do not always have the smallest F-statistics, but this is expected. Small between-group differences for highly variable genes are less significant and will produce smaller *F* statistics compared to the same magnitude of differences for less variable genes. While stability of genes is assessed relative to other genes, this metric still holds value in our analysis.

We validated stability of our genes across multiple independent datasets. To our knowledge, this was the first study to evaluate stability of genes across such a large and diverse set of data. Our evaluation of stability was performed across 14 datasets (with approximately 13000 samples) representing different biology (cancer, normal, and blood), collected by different consortia using different preparation protocols, measured with different instruments, processed using different pipelines and summarised using different metrics. Our set of stable genes were equally or more stable than other lists of stable genes in cancer and normal tissue and cell line datasets as shown by the smaller *M*-values (see Figure 2). Stability of all lists was poor and inconsistent in blood datasets, likely due to the genes being originally identified as stable in samples derived largely from solid tissue which is biologically very distinct from haematological and lymphoid tissues and cells. Genes with stable expression in blood could easily be identified by using our approach and appropriate data. An interesting observation was the stability of genes identified by Lin *et al.* (22) in label-free protein quantification data. Single-cell transcriptomic datasets suffer from the same problem of missing values as label free quantification, therefore genes determined to be stable in one will likely perform well in the other. Our list of stable genes and by extension other lists do not necessarily have poor stability in proteomic data, but they may be more difficult to measure. Stability is a prerequisite to rank preservation as evident from the low rank preservation in blood and proteomic data and high rank preservation in normal tissue. Though our list was identified using basal conditions, its stability was comparable to most other lists in data from perturbation experiments with the exception of data from the CMap project where control genes used in NanoString nCounter^®^ panels were more stable. Data from the CMap project is only partially measured therefore this observation may reflect the relative paucity of stable gene sets evaluated in this study in the directly measured LINCS1000 set. As such, this observation would need further affirmation in datasets from perturbation experiments where there are no measurement and prediction biases. Despite having better stability in CMap (Figure 4C), control genes in NanoString nCounter® panels had a narrower dynamic range of expression values (see Figure 4A), and therefore may not be the most ideal set of controls for normalisation and scoring of samples against gene expression signatures; this conclusion is supported by the analyses presented in Figure 4C and D.

Investigation into the functional roles of our proposed stable genes revealed that genes essential for cell survival tend to exhibit stable expression, however this is not universal. Further exploration of gene ontologies and other gene expression signatures from MSigDB revealed molecular processes involving RNA processing to be enriched in stably expressed genes. This observation indicates that these processes essential for cell survival are finely regulated and little room for error exists. Additionally, we showed that context/tissue-specific processes such as epithelial-mesenchymal transition were enriched with highly variable genes, as expected given the diverse changes associated with this phenotypic program (37). We used singscore (35) with stability ranks to enable this analysis, demonstrating that singscore can be used to assess enrichment using any ranked data.

Using the rank preservation property of stable genes, we developed a new molecular phenotyping method, *stingscore*, based on our original method (35). To date, this is the only approach capable of computing signature scores for single samples using a reduced set of transcriptomic measurements, such those obtained in a targeted study or RT-qPCR panel. We demonstrated that signature scores computed using the two methods and measurements are only strongly associated if they were computed using biologically meaningful gene signatures (see Figure 4B). Such signatures possess the power to discriminate samples therefore scores are correlated between approaches. In contrast, non-relevant signatures capture noise in the context of the biological problem being analysed. The choice of signatures used to analyse any biological problem should be motivated by prior knowledge of the biological system being analysed. Though scores generated using singscore and *stingscore* are correlated, they are not equivalent and there is generally an offset (see Figure 4B and C). This offset can be reduced if stable genes are selected in such a way that their distribution is close to uniform and they cover the entire range of expression values. Since our genes have this property the relative offset of scores is smaller than other lists (Figure 4C). Ranked sample order however is preserved and therefore scores retain their ability to differentiate clinically relevant groups such as those based on response to therapy (Figure 4D). This variation could be addressed by identifying other stable genes that could be used to calibrate scores. For instance, we could identify a stable gene set that represents the median score observed across all samples and adjust the score of each sample against the stable gene set score such that positive scores indicate concordance with a gene signature and vice versa. Nonetheless, we have shown that our method has clinical potential by demonstrating that patient samples can be scored against a relevant signature using as few as tens of transcript abundance measurements which could be acquired using lower throughput assays such as RT-qPCR.

More generally, our results demonstrate the potential for stable genes in clinical translation of biologically relevant gene sets through single sample gene expression signature scoring with a reduced panel of target genes. More sophisticated calibration methods and scoring methods capable of application to single samples, or small numbers of samples often resulting from clinical research, will be enabled by the ideas and methods presented in this work.

## CONCLUSION

Molecular profiling at the individual patient level is becoming increasingly useful in the clinic despite the lack of translation of such approaches. A wealth of molecular gene signatures such as those in the Molecular signatures database (MSigDB), remain unexplored in the clinic because of the requirement of whole transcriptome measurements imposed by most computational approaches that limit deployment in the context of regular pathology testing. In this study, we propose a novel cost-effective panel-based approach using stably expressed genes to assess gene expression signatures for individual patients in the clinic based on measurement of a substantially reduced number of genes (around two to three orders of magnitude fewer than whole transcriptome scale measurement). Since stable genes are used in this approach, no additional genes are required for data normalisation thus further saving costs of panel-based tests. This method will facilitate the adoption of gene expression signature analysis in a clinical context, thus allowing molecular profiling of a patient's disease, along with assessment of diagnostic/prognostic gene signatures, and assessment of signatures predictive of response to therapies.

## DATA AVAILABILITY

Methods developed in this study and the complete list of stable genes in human carcinomas are available in the R/Bioconductor package singscore (v1.8.0).

## Supplementary Material

gkaa802_Supplemental_Files

## References

[1] Narrandes S., Xu W. Gene expression detection assay for cancer clinical use. J. Cancer. 2018; 9:2249–2265. 30026820 10.7150/jca.24744 PMC6036716

[2] Marczyk M., Fu C., Lau R., Du L., Trevarton A.J., Sinn B.V., Gould R.E., Pusztai L., Hatzis C., Symmans W.F. The impact of RNA extraction method on accurate RNA sequencing from formalin-fixed paraffin-embedded tissues. BMC Cancer. 2019; 19:1189. 31805884 10.1186/s12885-019-6363-0 PMC6896723

[3] Paik S., Shak S., Tang G., Kim C., Baker J., Cronin M., Baehner F.L., Walker M.G., Watson D., Park T. et al. A multigene assay to predict recurrence of tamoxifen-treated, node-negative breast cancer. N. Engl. J. Med. 2004; 351:2817–2826. 15591335 10.1056/NEJMoa041588

[4] Gnant M., Filipits M., Greil R., Stoeger H., Rudas M., Bago-Horvath Z., Mlineritsch B., Kwasny W., Knauer M., Singer C. et al. Predicting distant recurrence in receptor-positive breast cancer patients with limited clinicopathological risk: using the PAM50 Risk of recurrence score in 1478 postmenopausal patients of the ABCSG-8 trial treated with adjuvant endocrine therapy alone. Ann. Oncol. 2014; 25:339–345. 24347518 10.1093/annonc/mdt494

[5] Finotello F., Mayer C., Plattner C., Laschober G., Rieder D., Hackl H., Krogsdam A., Loncova Z., Posch W., Wilflingseder D. et al. Molecular and pharmacological modulators of the tumor immune contexture revealed by deconvolution of RNA-seq data. Genome Med. 2019; 11:34. 31126321 10.1186/s13073-019-0638-6 PMC6534875

[6] Wang X., Sun Z., Zimmermann M.T., Bugrim A., Kocher J.P. Predict drug sensitivity of cancer cells with pathway activity inference. BMC Med Genomics. 2019; 12:15. 30704449 10.1186/s12920-018-0449-4 PMC6357358

[7] Liberzon A., Subramanian A., Pinchback R., Thorvaldsdóttir H., Tamayo P., Mesirov J.P. Molecular signatures database (MSigDB) 3.0. Bioinformatics. 2011; 27:1739–1740. 21546393 10.1093/bioinformatics/btr260 PMC3106198

[8] Subramanian A., Tamayo P., Mootha V.K., Mukherjee S., Ebert B.L., Gillette M.A., Paulovich A., Pomeroy S.L., Golub T.R., Lander E.S. et al. Gene set enrichment analysis: a knowledge-based approach for interpreting genome-wide expression profiles. Proc. Natl. Acad. Sci. U.S.A. 2005; 102:15545–15550. 16199517 10.1073/pnas.0506580102 PMC1239896

[9] Pennock N.D., Jindal S., Horton W., Sun D., Narasimhan J., Carbone L., Fei S.S., Searles R., Harrington C.A., Burchard J. et al. RNA-seq from archival FFPE breast cancer samples: molecular pathway fidelity and novel discovery. BMC Med Genomics. 2019; 12:195. 31856832 10.1186/s12920-019-0643-z PMC6924022

[10] Vandesompele J., De Preter K., Pattyn F., Poppe B., Van Roy N., De Paepe A., Speleman F. Accurate normalization of real-time quantitative RT-PCR data by geometric averaging of multiple internal control genes. Genome Biol. 2002; 3:doi:10.1186/gb-2002-3-7-research0034. 10.1186/gb-2002-3-7-research0034 PMC126239 12184808

[11] Jacob L., Gagnon-Bartsch J.A., Speed T.P. Correcting gene expression data when neither the unwanted variation nor the factor of interest are observed. Biostatistics. 2016; 17:16–28. 26286812 10.1093/biostatistics/kxv026 PMC4679071

[12] Baker S.C., Bauer S.R., Beyer R.P., Brenton J.D., Bromley B., Burrill J., Causton H., Conley M.P., Elespuru R., Fero M. et al. The external RNA controls Consortium: a progress report. Nat. Methods. 2005; 2:731–734. 16179916 10.1038/nmeth1005-731

[13] Grun D., van Oudenaarden A. Design and analysis of Single-Cell sequencing experiments. Cell. 2015; 163:799–810. 26544934 10.1016/j.cell.2015.10.039

[14] Molania R., Gagnon-Bartsch J.A., Dobrovic A., Speed T.P. A new normalization for Nanostring nCounter gene expression data. Nucleic Acids Res. 2019; 47:6073–6083. 31114909 10.1093/nar/gkz433 PMC6614807

[15] Tung P.Y., Blischak J.D., Hsiao C.J., Knowles D.A., Burnett J.E., Pritchard J.K., Gilad Y. Batch effects and the effective design of single-cell gene expression studies. Sci. Rep. 2017; 7:39921. 28045081 10.1038/srep39921 PMC5206706

[16] Chapman J.R., Waldenstrom J. With reference to reference genes: a systematic review of endogenous controls in gene expression studies. PLoS One. 2015; 10:e0141853. 26555275 10.1371/journal.pone.0141853 PMC4640531

[17] Chen L., Jin Y., Wang L., Sun F., Yang X., Shi M., Zhan C., Shi Y., Wang Q. Identification of reference genes and miRNAs for qRT-PCR in human esophageal squamous cell carcinoma. Med. Oncol. 2017; 34:2. 27889881 10.1007/s12032-016-0860-7

[18] Chim S.S.C., Wong K.K.W., Chung C.Y.L., Lam S.K.W., Kwok J.S.L., Lai C.Y., Cheng Y.K.Y., Hui A.S.Y., Meng M., Chan O.K. et al. Systematic selection of reference genes for the normalization of circulating RNA transcripts in pregnant women based on RNA-Seq data. Int. J. Mol. Sci. 2017; 18:1709. 10.3390/ijms18081709 PMC5578099 28777335

[19] Hoang V.L.T., Tom L.N., Quek X.C., Tan J.M., Payne E.J., Lin L.L., Sinnya S., Raphael A.P., Lambie D., Frazer I.H. et al. RNA-seq reveals more consistent reference genes for gene expression studies in human non-melanoma skin cancers. PeerJ. 2017; 5:e3631. 28852586 10.7717/peerj.3631 PMC5572537

[20] Eisenberg E., Levanon E.Y. Human housekeeping genes, revisited. Trends Genet. 2013; 29:569–574. 23810203 10.1016/j.tig.2013.05.010

[21] Krasnov G.S., Kudryavtseva A.V., Snezhkina A.V., Lakunina V.A., Beniaminov A.D., Melnikova N.V., Dmitriev A.A. Pan-Cancer Analysis of TCGA data revealed promising reference genes for qPCR normalization. Front Genet. 2019; 10:97. 30881377 10.3389/fgene.2019.00097 PMC6406071

[22] Lin Y., Ghazanfar S., Strbenac D., Wang A., Patrick E., Lin D.M., Speed T., Yang J.Y.H., Yang P. Evaluating stably expressed genes in single cells. Gigascience. 2019; 8:giz106. 31531674 10.1093/gigascience/giz106 PMC6748759

[23] Hong F., Breitling R. A comparison of meta-analysis methods for detecting differentially expressed genes in microarray experiments. Bioinformatics. 2008; 24:374–382. 18204063 10.1093/bioinformatics/btm620

[24] Wang C., Taciroglu A., Maetschke S.R., Nelson C.C., Ragan M.A., Davis M.J. mCOPA: analysis of heterogeneous features in cancer expression data. J Clin Bioinforma. 2012; 2:22. 23216803 10.1186/2043-9113-2-22 PMC3553066

[25] Robinson M.D., Oshlack A. A scaling normalization method for differential expression analysis of RNA-seq data. Genome Biol. 2010; 11:R25. 20196867 10.1186/gb-2010-11-3-r25 PMC2864565

[26] Bhuva D.D., Foroutan M., Xie Y., Lyu R., Cursons J., Davis M.J. Using singscore to predict mutation status in acute myeloid leukemia from transcriptomic signatures. F1000Res. 2019; 8:776. 31723419 10.12688/f1000research.19236.1 PMC6844140

[27] Smirnov P., Safikhani Z., El-Hachem N., Wang D., She A., Olsen C., Freeman M., Selby H., Gendoo D.M., Grossmann P. et al. PharmacoGx: an R package for analysis of large pharmacogenomic datasets. Bioinformatics. 2016; 32:1244–1246. 26656004 10.1093/bioinformatics/btv723

[28] Subramanian A., Narayan R., Corsello S.M., Peck D.D., Natoli T.E., Lu X., Gould J., Davis J.F., Tubelli A.A., Asiedu J.K. et al. A next generation connectivity Map: L1000 platform and the first 1,000,000 profiles. Cell. 2017; 171:1437–1452. 29195078 10.1016/j.cell.2017.10.049 PMC5990023

[29] Foroutan M., Cursons J., Hediyeh-Zadeh S., Thompson E.W., Davis M.J. A transcriptional program for detecting TGFbeta-Induced EMT in cancer. Mol. Cancer Res. 2017; 15:619–631. 28119430 10.1158/1541-7786.MCR-16-0313

[30] Barretina J., Caponigro G., Stransky N., Venkatesan K., Margolin A.A., Kim S., Wilson C.J., Lehar J., Kryukov G.V., Sonkin D. et al. The Cancer Cell Line Encyclopedia enables predictive modelling of anticancer drug sensitivity. Nature. 2012; 483:603–607. 22460905 10.1038/nature11003 PMC3320027

[31] Daemen A., Griffith O.L., Heiser L.M., Wang N.J., Enache O.M., Sanborn Z., Pepin F., Durinck S., Korkola J.E., Griffith M. et al. Modeling precision treatment of breast cancer. Genome Biol. 2013; 14:R110. 24176112 10.1186/gb-2013-14-10-r110 PMC3937590

[32] Schmiedel B.J., Singh D., Madrigal A., Valdovino-Gonzalez A.G., White B.M., Zapardiel-Gonzalo J., Ha B., Altay G., Greenbaum J.A., McVicker G. et al. Impact of genetic polymorphisms on human immune cell gene expression. Cell. 2018; 175:1701–1715. 30449622 10.1016/j.cell.2018.10.022 PMC6289654

[33] Dempster J.M., Rossen J., Kazachkova M., Pan J., Kugener G., Root D.E., Tsherniak A. Extracting biological insights from the project achilles Genome-Scale CRISPR screens in cancer cell lines. 2019; bioRxiv doi:31 July 2019, preprint: not peer reviewed 10.1101/720243.

[34] Meyers R.M., Bryan J.G., McFarland J.M., Weir B.A., Sizemore A.E., Xu H., Dharia N.V., Montgomery P.G., Cowley G.S., Pantel S. et al. Computational correction of copy number effect improves specificity of CRISPR-Cas9 essentiality screens in cancer cells. Nat. Genet. 2017; 49:1779–1784. 29083409 10.1038/ng.3984 PMC5709193

[35] Foroutan M., Bhuva D.D., Lyu R., Horan K., Cursons J., Davis M.J. Single sample scoring of molecular phenotypes. BMC Bioinformatics. 2018; 19:404. 30400809 10.1186/s12859-018-2435-4 PMC6219008

[36] Bhuva D.D., Cursons J., Smyth G.K., Davis M.J. Differential co-expression-based detection of conditional relationships in transcriptional data: comparative analysis and application to breast cancer. Genome Biol. 2019; 20:236. 31727119 10.1186/s13059-019-1851-8 PMC6857226

[37] Cursons J., Pillman K.A., Scheer K.G., Gregory P.A., Foroutan M., Hediyeh-Zadeh S., Toubia J., Crampin E.J., Goodall G.J., Bracken C.P. et al. Combinatorial targeting by microRNAs co-ordinates post-transcriptional control of EMT. Cell Syst. 2018; 7:77–91. 30007539 10.1016/j.cels.2018.05.019

[38] Cursons J., Souza-Fonseca-Guimaraes F., Foroutan M., Anderson A., Hollande F., Hediyeh-Zadeh S., Behren A., Huntington N.D., Davis M.J. A gene signature predicting natural killer cell infiltration and improved survival in melanoma patients. Cancer Immunol. Res. 2019; 7:1162–1174. 31088844 10.1158/2326-6066.CIR-18-0500

[39] Consortium S.M.-I. A comprehensive assessment of RNA-seq accuracy, reproducibility and information content by the Sequencing Quality Control Consortium. Nat. Biotechnol. 2014; 32:903–914. 25150838 10.1038/nbt.2957 PMC4321899

[40] Whitfield M.L., George L.K., Grant G.D., Perou C.M. Common markers of proliferation. Nat. Rev. Cancer. 2006; 6:99–106. 16491069 10.1038/nrc1802

[41] Chang J.C., Wooten E.C., Tsimelzon A., Hilsenbeck S.G., Gutierrez M.C., Elledge R., Mohsin S., Osborne C.K., Chamness G.C., Allred D.C. et al. Gene expression profiling for the prediction of therapeutic response to docetaxel in patients with breast cancer. Lancet. 2003; 362:362–369. 12907009 10.1016/S0140-6736(03)14023-8

[42] Deng K., Han S.M., Li K.J., Liu J.S. Bayesian aggregation of order-based rank data. J. Am. Stat. Assoc. 2014; 109:1023–1039.

[43] Law C.W., Chen Y., Shi W., Smyth G.K. voom: precision weights unlock linear model analysis tools for RNA-seq read counts. Genome Biol. 2014; 15:R29. 24485249 10.1186/gb-2014-15-2-r29 PMC4053721

[44] Colaprico A., Silva T.C., Olsen C., Garofano L., Cava C., Garolini D., Sabedot T.S., Malta T.M., Pagnotta S.M., Castiglioni I. et al. TCGAbiolinks: an R/Bioconductor package for integrative analysis of TCGA data. Nucleic Acids Res. 2016; 44:e71. 26704973 10.1093/nar/gkv1507 PMC4856967

[45] Uhlen M., Fagerberg L., Hallstrom B.M., Lindskog C., Oksvold P., Mardinoglu A., Sivertsson A., Kampf C., Sjostedt E., Asplund A. et al. Proteomics. Tissue-based map of the human proteome. Science. 2015; 347:1260419. 25613900 10.1126/science.1260419

[46] Zhang B., Wang J., Wang X., Zhu J., Liu Q., Shi Z., Chambers M.C., Zimmerman L.J., Shaddox K.F., Kim S. et al. Proteogenomic characterization of human colon and rectal cancer. Nature. 2014; 513:382–387. 25043054 10.1038/nature13438 PMC4249766

[47] FANTOM Consortium and the RIKEN PMI and CLST (DGT) Forrest A.R., Kawaji H., Rehli M., Baillie J.K., de Hoon M.J., Haberle V., Lassmann T., Kulakovskiy I.V., Lizio M. et al. A promoter-level mammalian expression atlas. Nature. 2014; 507:462–470. 24670764 10.1038/nature13182 PMC4529748

[48] Lizio M., Abugessaisa I., Noguchi S., Kondo A., Hasegawa A., Hon C.C., de Hoon M., Severin J., Oki S., Hayashizaki Y. et al. Update of the FANTOM web resource: expansion to provide additional transcriptome atlases. Nucleic Acids Res. 2019; 47:D752–D758. 30407557 10.1093/nar/gky1099 PMC6323950

[49] Lizio M., Harshbarger J., Shimoji H., Severin J., Kasukawa T., Sahin S., Abugessaisa I., Fukuda S., Hori F., Ishikawa-Kato S. et al. Gateways to the FANTOM5 promoter level mammalian expression atlas. Genome Biol. 2015; 16:22. 25723102 10.1186/s13059-014-0560-6 PMC4310165

[50] Linsley P.S., Speake C., Whalen E., Chaussabel D. Copy number loss of the interferon gene cluster in melanomas is linked to reduced T cell infiltrate and poor patient prognosis. PLoS One. 2014; 9:e109760. 25314013 10.1371/journal.pone.0109760 PMC4196925

[51] Monaco G., Lee B., Xu W., Mustafah S., Hwang Y.Y., Carre C., Burdin N., Visan L., Ceccarelli M., Poidinger M. et al. RNA-Seq Signatures Normalized by mRNA Abundance Allow Absolute Deconvolution of Human Immune Cell Types. Cell Rep. 2019; 26:1627–1640. 30726743 10.1016/j.celrep.2019.01.041 PMC6367568

